# Age variation and sexual dimorphism in the sixteen diagnostic clusters of risk factors for the metabolic syndrome

**DOI:** 10.1007/s10389-012-0490-2

**Published:** 2012-01-26

**Authors:** S. S. Sun, Roy Sabo, S. Arslanian, Ruishan Wu, Cynthia Sabo

**Affiliations:** 1 Department of Biostatistics, School of Medicine, Virginia Commonwealth University, 830 East Main Street, P.O. Box 980032, Richmond, VA 23298-0032 USA; 2 Department of Pediatrics, University of Pittsburgh School of Medicine, CHL 7628, Pittsburgh, PA 15213 USA; 3 Takeda Global Research and Development, One Takeda Parkway, Deerfield, IL 60015 USA

**Keywords:** Metabolic syndrome, Triad, Sexual dimorphism

## Abstract

**Objective:**

To document age- and sex-related differences in the 16 phenotypes of risk factors for the metabolic syndrome (MS) among adults in the Fels Longitudinal Study (FLS).

**Methods:**

Data on risk factors for the MS were analyzed in 471 white men and 503 white women in the FLS. We used the Cochran-Armitage test to compare age- and sex-related differences in the prevalence of the 16 diagnostic clusters of positive risk factors.

**Results:**

Of the 974 subjects, 238 were found to meet diagnostic criteria for 15 of a possible 16 phenotypes of the MS. The prevalence of the MS was four times greater in subjects older than 40 years than in subjects 20–40 years old. Older subjects had more risk factors exceeding criterion values than younger subjects. Among those who met three-to-five criteria for the MS, younger subjects were more likely to have dyslipidemia, less likely to have high blood pressure (HBP), and two times less likely to have impaired fasting plasma glucose (IFG) than subjects 40+ years old. Older men were more likely than older women to have HBP and IFG. . We found that if one of the five risk factors reaches a criterion value, the values for the other four risk factors move closer to their own diagnostic criterion values in apparent synchrony.

**Conclusions:**

Subjects 40+ years old are four times likelier to have the MS than younger subjects, and older men are at higher risk than older women. The mean values for each of the five risk factors get progressively worse as the number of risk factors meeting diagnostic criteria increases. Therefore, when one factor is found to meet its diagnostic criterion, levels of the other four risk factors should be measured. The different phenotypic patterns that comprise the MS should prompt clinicians to target specific risk factors for prevention or treatment. Certain phenotypes were found more commonly in women and certain others more commonly in men. Similarly, certain phenotypes were found more commonly in older than in younger age groups. These age- and sex-specific phenotypes should help clinicians to identify subjects at highest risk for certain risk factors and to initiate specifically tailored preventive and therapeutic interventions. Our observations should also stimulate clinical investigators and epidemiologists to ascertain what factors determine the sex and age specificity of certain phenotypes of the MS.

## Introduction

The Third Report of the National Cholesterol Education Program (NCEP) Adult Treatment Panel III (ATP III) on Detection, Evaluation, and Treatment of High Blood Cholesterol in Adults provides a working definition of the MS (Adult Treatment Panel III 2001). The ATP III defines the MS as a cluster of three, four, or five risk factors that exceed criterion values, namely: waist circumference >102 cm for men and >88 cm for women; systolic blood pressure ≥130 and/or diastolic blood pressure ≥85 mm Hg; fasting plasma triglyceride level ≥150 mg/dl; fasting plasma HDL-cholesterol level <40 mg/dl for men and <50 mg/dl for women; and fasting plasma glucose concentration >100 mg/dl.

Using the NCEP ATP III criteria, Ford et al. (2002) reported that approximately 24% of US adults have the MS and that its prevalence is age-dependent and sexually dimorphic. The prevalence of the MS increases from 7% in the third decade to 44% in the seventh decade. While the overall prevalence differs little between men (24%) and women (23%), the frequency distribution of risk factors that contribute to the metabolic syndrome differs greatly between men and women. Women are more likely than men to meet the criteria for abdominal obesity and low levels of fasting HDL-cholesterol, while men are more likely than women to meet the criteria for hypertriglyceridemia, HBP and IFG (Ford et al. 2002; Park et al. 2004).

The MS comprises 16 possible clusters of the five diagnostic risk factors taken in combinations of three, four or five. In the Fels Longitudinal Study (FLS) data, several of the 16 clusters are more common than others. Information on the prevalence by age and sex of the 16 clusters of risk factors that define the syndrome may elucidate the origins of the MS and may sharpen predictions for the metabolic and cardiovascular consequences of the MS. This information may lead to improvements in the clinical management of patients who meet one or two but not three of the ATP III diagnostic criteria for the MS.

We analyzed data from the FLS to examine age- and sex-related differences in the frequency distribution of the 16 possible clusters of three or more of the five risk factors that define the MS. We conducted the study using both the NCEP ATP III criterion of 110 mg/dl and the ADA recommended criterion of 100 mg/dl for impaired fasting plasma glucose in order to elucidate the diagnostic implications of the lower threshold (Genuth et al. 2003).

## Subjects and methods

### Study sample

The study sample consists of 471 white men and 503 white women participants in the FLS. Initiated in 1929, the FLS is the world’s largest and oldest longitudinal study of human growth and body composition (Roche 1992). Approximately 8% of FLS participants have been lost to follow-up over the eight decades of observation and 16% have died, but their body composition and cardiovascular data at their last visit did not differ from those who remain active in the study. Childhood measurements made on FLS participants from birth include weight, height, skinfold thicknesses, arm, head and waist circumferences, and blood pressure. These data are recorded during regular examinations at 1, 3, 6, 9, and 12 months, then semi-annually to 18 years, and biennially thereafter. In 1976 examinations of body composition by hydrodensitometry were included, as well as determinations of fasting plasma lipids, lipoproteins, glucose, and insulin concentrations when the participants turned 8 years of age.

In this study of 974 FLS participants, data were analyzed on waist circumference, systolic blood pressure, and levels of fasting plasma HDL-cholesterol, triglycerides, and glucose. Waist circumference was measured using standardized procedures similar to those recommended at the Airlie Consensus Conference (Lohman et al. 1988). Blood pressure was measured according to the standards of the Second NHLBI Task Force on Blood Pressure Control in Children and the update of that report by the National High Blood Pressure Education Program (Falkner et al. 1996). Fasting plasma levels of triglycerides, HDL-cholesterol and total cholesterol were measured (NHLBI 1974) at the Medical Research Laboratory in Cincinnati, OH. Measurements of fasting plasma glucose and insulin concentrations were also performed at the Medical Research Laboratory using a standard insulin radioimmunoassay (Linco Research, St. Louis, MO) and a standard colorimetric method for measurement of plasma glucose. All procedures were approved by the Institutional Review Boards of Virginia Commonwealth University and Wright State University.

### Statistical analysis

The prevalence of the MS was determined for men and women in two age groups: 20–40 years and greater than 40 years. The prevalences of zero, one or two risk factors exceeding criteria were also ascertained for comparative analysis. Since the diagnosis of the MS requires that at least three of five risk factors exceed criterion values, it is possible to have 10 diagnostic combinations of three risk factors, five diagnostic combinations of four risk factors, and one combination of all five risk factors. The frequency distributions of the 16 combinations of risk factors that define the MS were computed separately for both sexes and both age groups. In order to assess the degree of deviation from non-diagnostic values, means and standard deviations for the five risk factors were compared between adults with the MS and adults with zero, one, or two risk factors that exceeded diagnostic criteria. The effects of age and gender on the risk factor(s) of the MS were assessed using an analysis of variance (ANOVA) model with fixed effects for age, gender, and age-by-gender interaction. The Cochran-Armitage trend test was applied to compare age- and gender-related differences in the prevalence of the MS and the 16 possible diagnostic clusters of risk factors. Analyses were performed using the Statistical Analysis System (SAS Version 9.2 SAS Institute).

## Results

### Sample characteristics

**Fig. 1:**
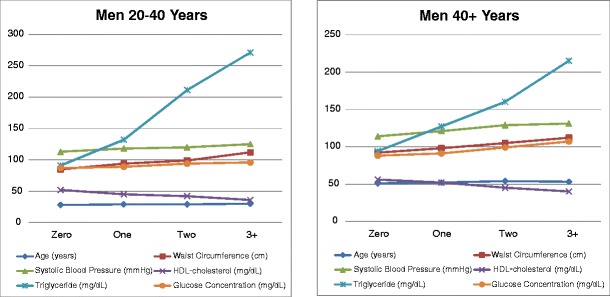
Means values for risk factors for metabolic syndrome in male FLS subjects according to number of risk factors that exceed ATP III diagnostic criteria

**Fig. 2:**
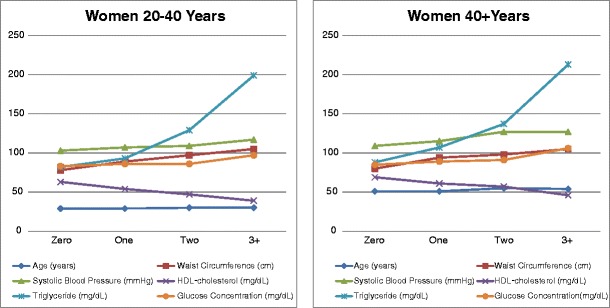
Means values for risk factors for metabolic syndrome in female FLS subjects according to number of risk factors that exceed ATP III diagnostic criteria

We found significant age and sex differences in the mean values of the five risk factors for the MS (Table 1). In general, the age-related difference in mean values for the risk factors increases with the number of risk factors meeting the ATP III diagnostic criteria for the MS. Figures 1 and 2 show that within both age groups the mean value for each risk factor approaches the diagnostic criterion value for that risk factor in direct proportion to the number of risk factors that meet diagnostic criteria, from zero to three or more. For instance, if one of the five risk factors reaches a diagnostic criterion value, the mean values for each of the other four risk factors move closer to their respective diagnostic criterion values in apparent synchrony, and if two risk factors reach their diagnostic criterion values, the mean values for the other three risk factors move even closer to their respective diagnostic criterion values, again in apparent synchrony. This effect is most pronounced for the elevated fasting plasma triglycerides.

**Table 1:** Means and standard deviations (SD) of values for risk factors for metabolic syndrome in FLS subjects according to number of risk factors that exceed ATP III diagnostic criteria

	Zero risk factors			One risk factors			Two risk factors			3+ riskfactors		
	*N*	Mean	SD	*N*	Mean	SD	*N*	Mean	SD	*N*	Mean	SD
Men 20–40 years												
Age (years)	231	28	4.3	81	29	4.4	47	29	4.8	33	30	4.4
Waist circumference (cm)	164	85^**, ****^	7.1	73	94^**, ****^	13.3	47	99^*, ***^	14.2	33	112^***^	14.7
Systolic blood pressure (mmHg)	231	113^****^	7.5	81	118^****^	9.9	47	120^** ****^	10.5	33	125^**, g^	9.5
HDL-cholesterol (mg/dl)	151	52^**, ****^	8.5	70	45^**, ****^	10.2	45	42^***^	9.7	33	36^**, ****^	5.3
Triglyceride (mg/dl)	151	91^****^	28.4	70	132^****^	69.9	45	211^*, ****^	183.8	33	271^*, ***^	161.3
Glucose concentration (mg/dl)	108	87^**, ****^	7.4	58	89^*, ***^	7.9	40	94^*, ****^	8.5	31	96^*^	11.0
Men 40+ years												
Age (years)	75	51	7.0	61	52	10.0	64	54	8.7	102	53	9.1
Waist circumference (cm)	75	92^**, ****^	6.4	59	98^**, **^	9.0	64	105^*, ***^	9.7	102	112^***^	11.2
Systolic blood pressure (mmHg)	75	114^****^	8.1	61	121^***^	14.2	64	129^**^	13.2	102	131^**, ***^	11.1
HDL-cholesterol (mg/dl)	69	56^**, ****^	11.0	58	52^**, ****^	11.8	64	45^****^	8.7	102	40^**, ****^	6.8
Triglyceride (mg/dl)	69	94^****^	27.2	58	127^****^	51.1	64	160^*^	72.8	102	215^*^	85.1
Glucose concentration (mg/dl)	71	88^*, ****^	5.8	58	91^*, ***^	9.6	64	99^*, ****^	19.8	99	107^*^	21.3
Women 20–40 years												
Age (years)	242	29	4.1	123	29	4.7	36	30	4.9	23	30	5.4
Waist circumference (cm)	162	78^*, ****^	4.9	121	89^**, ****^	11.2	36	97^*, ***^	11.8	23	105^***^	11.1
Systolic blood pressure (mmHg)	241	103^** ****^	7.2	122	107^**, ****^	8.8	36	109^** ****^	8.8	23	117^**, ***^	11.6
HDL-cholesterol (mg/dl)	149	63^**,****^	10.0	118	54^**, ****^	10.5	36	47^**, ***^	7.1	23	39^**, ****^	6.4
Triglyceride (mg/dl)	149	82^****^	24.2	118	93^***^	34.5	36	129^**^	65.6	23	199^***^	66.7
Glucose Concentration (mg/dl)	118	83^**, ****^	8.1	93	86^*, ***^	11.6	27	86^*, ****^	6.4	23	97^*^	25.6
Women 40+ years												
Age (years)	92	51	7.3	101	51	8.4	62	55	10.8	80	54	10.0
Waist circumference (cm)	92	80^**, ****^	5.0	101	94^**, ****^	10.5	62	98^*, ***^	12.6	80	105^***^	13.0
Systolic blood pressure (mmHg)	92	109^** ****^	9.5	101	115^**, ***^	14.5	62	127^**^	15.6	80	127^**, g^	13.7
HDL-cholesterol (mg/dl)	87	69^**, ****^	12.0	99	61^**, ****^	10.9	58	57^**, **^	12.5	80	46^**, ****^	9.1
Triglyceride (mg/dl)	87	88^****^	24.2	99	107^****^	32.7	58	137	41.1	80	213	78.6
Glucose Concentration (mg/dl)	89	85^*, ****^	6.2	99	89^*, ***^	6.1	60	91^*, ****^	10.0	80	106^*^	29.0

Note: The statistical analysis was based on with fixed effects for age group, gender, and age group-by-gender interaction for each metabolic syndrome risk factor

*Significant age different *p* < 0.05

**Significant age different *p* < 0.001

***Significant gender different *p* < 0.05

****Significant gender different *p* < 0.001

### Clustering of components of the MS

#### Younger vs. older subjects

**Fig. 3:**
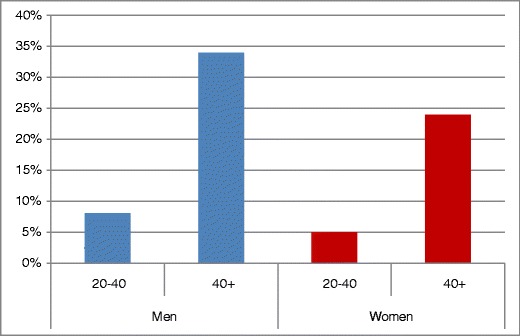
Prevalence of FLS subjects whose risk factor values exceed three or more ATP III criteria

The prevalence of the MS was four times as great among the older vs. the younger age groups (*p* < 0.0001). Among both men and women, there is a significant difference in the prevalence of MS between age groups—*p* < 0.0001 (Table 2; Fig. 3). Older subjects tended to be more obese, dyslipidemic, and hypertensive than younger ones (Table 1). Men and women older than 40 years with the MS also had a significantly greater number of risk factors that exceed diagnostic criterion values than men and women 20–40 years old, according to the Cochrane-Armitage Trend Test (*p* < 0.05 (Table 2). Overall, the men had a prevalence of the MS 50% greater than the women (*p* = 0.0025); however, when analyzed by age group, the prevalence differs significantly between men and women in the older (*p* = 0.0058) but not the younger age group—*p* = 0.091 (Table 2; Fig. 3).

**Table 2:** Numbers and prevalence^*^ of FLS subjects whose risk factor values exceed ATP III criteria for zero to five risk factors

	20–40 years		40+ years	
	Men	Women	Men	Women
Non-metabolic syndrome^**, ***^	359 (92%)^**^	401 (95%)^**^	200 (66%)^**, ***^	255 (76%)^**, ***^
Zero risk factors	231 (59%)	242 (57%)	75 (25%)	92 (27%)
One risk factor	81 (21%)	123 (29%)	61 (20%)	101 (30%)
Two risk factors	47 (12%)	36 (8%)	64 (21%)	62 (19%)
Metabolic syndrome^**, ***^	33 (8%)^**^	23 (5%)^**^	102 (34%)^**, ***^	80 (24%)^**, ***^
Three risk factors	23 (6%)	17 (4%)	56 (19%)	46 (14%)
Four risk factors	8 (2%)	5 (1%)	33 (11%)	25 (7%)
Five risk factors	2 (1%)	1 (0%)	13 (4%)	9 (3%)

Note 1: Prevalence: Number of subjects in each age-by-sex-by risk factor category divided by the total number of subjects in each column, expressed as a percentage

Note 2: Cochran-Armitage trend test was used to analyze the underlying trend between age groups and gender

^*^Significant age different *p* < 0.05

^**^Significant age different *p* < 0.001

^***^Significant gender different *p* < 0.05

#### Subjects meeting three criteria for the MS

**Fig. 4:**
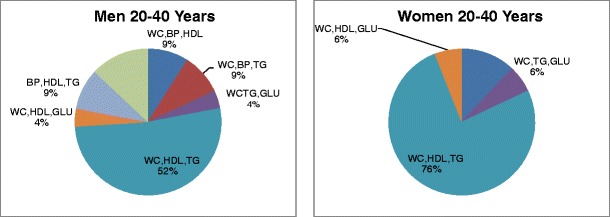
Triad phenotypes of metabolic syndrome in FLS participants 20–40 years of age

**Fig. 5:**
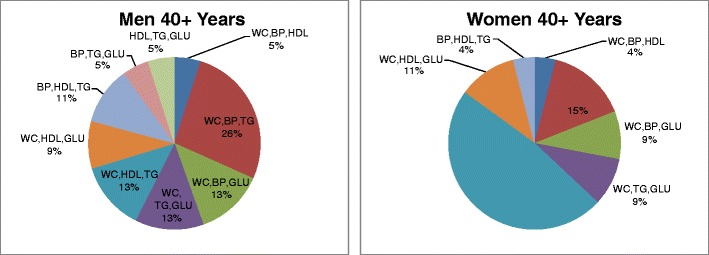
Triad phenotypes of metabolic syndrome in FLS participants 40+ years of age

Among 20–40 year-old subjects with the MS, 76% of the women and 52% of the men met criteria for the diagnostic triad of increased waist circumference, low level of fasting plasma HDL-cholesterol, and elevated level of fasting plasma triglycerides (Table 3; Fig. 4). Among subjects older than 40 years with the MS, 48% of women met criteria for the same diagnostic triad compared to 13% of men (Table 3; Fig. 5). The smaller percentages of older women and men who met criteria for this triad compared to younger women and men may be explained by the greater numbers of older women and men meeting diagnostic criteria for eight of the other nine diagnostic triads. For example, men 20–40 years old with the MS met criteria for seven of the 10 possible diagnostic triads, whereas men older than 40 years with three positive risk factors met criteria for nine of the 10 diagnostic triads. Women 20–40 years old with the MS met criteria for only four of the 10 diagnostic triads, while women older than 40 years with three positive risk factors met criteria for seven of the 10 diagnostic triads (Table 3). It is interesting to note that the risk factors for the MS cluster both with and without increased waist circumference. This observation indicates that the clusters of risk factors that define the MS are not necessarily driven by centripetal obesity.

**Table 3:** Numbers and proportions (%) of clusters of risk factors diagnostic of metabolic syndrome in FLS subjects by age group and sex

	20–40 years		40+ years	
	Men	Women	Men	Women
Three risk factors				
WC, BP, HDL	2 (9%)	2 (12%)	3 (5%)	2 (4%)
WC, BP, TG	2 (9%)		15 (27%)	7 (15%)
WC, BP, GLU			7 (13%)	4 (9%)
WC, TG, GLU	1 (4%)	1 (6%)	7 (13%)	4 (9%)
WC, HDL, TG	12 (52%)	13 (76%)	7 (13%)	22 (48%)
WC, HDL, GLU	1 (4%)	1 (6%)	5 (9%)	5 (11%)
BP, HDL, TG	2 (9%)		6 (11%)	2 (4%)
BP, TG, GLU			3 (5%)	
HDL, TG, GLU	3 (13%)		3 (5%)	
HDL, BP, GLU				
Total	23	17	56	46
Four risk factors				
WC, BP, HDL,TG	6 (75%)	2 (40%)	12 (36%)	8 (32%)
WC, BP, HDL, GLU		1 (20%)	3 (9%)	3 (12%)
WC, BP, TG, GLU			6 (18%)	3 (12%)
WC, HDL, TG, GLU	1 (13%)	2 (40%)	11 (33%)	11 (44%)
BP, HDL, TG, GLU	1 (13%)		1 (3%)	
Total	8	5	33	25
Five risk factors				
WC, BP, HDL, TG, GLU	2	1	13	9
Total number of adults with metabolic syndrome	33	23	102	80

*WC* waist circumference; *SBP* systolic blood pressure; *HDL* fasting plasma high density lipoprotein cholesterol; *TG* fasting plasma triglycerides; *GLU* impaired fasting plasma glucose concentration

#### Subjects meeting two criteria for the MS

Subjects who meet two criteria for a diagnosis of the MS are, presumably, at greater risk for meeting three or more diagnostic criteria than subjects who meet zero criteria. Therefore, we analyzed the frequency distribution of the five risk factors in subjects meeting two criteria according to age and sex. There are 10 combinations of any two of the five risk factors that meet criteria for the MS. Women 20–40 years old met criteria for five of the 10 possible dyads, whereas women older than 40 years met criteria for eight of the ten dyads (Table 4). Men 20–40 years old met criteria for nine of the 10 dyads, and men older than 40 years met criteria for all ten. Among 47 men between 20 and 40 years old who met two, but not more, criterion values, the most prevalent dyad, was a low level of fasting plasma HDL-cholesterol and an elevated level of fasting plasma triglycerides found in 32% of these men. Among the 64 men older than 40 years, the most prevalent dyad, found in 33% of them, was increased waist circumference and HBP (Table 4). Among the 36 women between the ages of 20 and 40 years who met two criteria, the dyad of increased waist circumference and low level of fasting plasma HDL-cholesterol was the most prevalent, found in 64% of these women. Three dyads with increased waist circumference accounted for 80% of the dyads that met criterion values, one coupled with HBP; one coupled with increased fasting plasma triglycerides; and one coupled with a low level of fasting plasma HDL-cholesterol. These dyads were found in 31, 24, and 23%, respectively, of the women over 40 years with two positive risk factors. (Table 4).

**Table 4:** Numbers and proportions (%) of zero, one, or combinations of two risk factors exceeding ATP III criteria for metabolic syndrome in FLS subjects adults 20 years and older by age group and sex

	20–40 years		40+ years	
	Men	Women	Men	Women
Two risk factors				
WC, BP	7 (15%)	1 (3%)	21 (33%)	19 (31%)
WC, TG	7 (15%)	5 (14%)	7 (11%)	15 (24%)
WC, HDL	4 (9%)	23 (64%)	3 (5%)	14 (23%)
WC, GLU	2 (4%)	1 (3%)	7 (11%)	5 (8%)
BP, HDL			2 (3%)	4 (6%)
BP, TG	3 (6%)		6 (9%)	3 (5%)
BP, GLU	1 (2%)		5 (8%)	1 (2%)
HDL, TG	15 (32%)	6 (17%)	7 (11%)	
HDL, GLU	3 (6%)		1 (2%)	
TG, GLU	5 (11%)		5 (8%)	1 (2%)
Total	47	36	64	62
One risk factor				
WC	17 (21%)	62 (50%)	15 (25%)	74 (73%)
BP	16 (20%)	3 (2%)	19 (31%)	9 (9%)
HDL	22 (27%)	45 (37%)	4 (7%)	9 (9%)
TG	21 (26%)	9 (7%)	14 (23%)	8 (8%)
GLU	5 (6%)	4 (3%)	9 (15%)	1 (1%)
Total	81	123	61	101
Zero risk factors	231	242	75	92
Total number of adults without metabolic syndrome	359	401	200	255

*WC* waist circumference; *SBP* systolic blood pressure; *HDL* fasting plasma high density lipoprotein cholesterol; *TG* fasting plasma triglycerides; *GLU* impaired fasting plasma glucose concentration

#### Subjects meeting one criterion for the MS

Among the eighty-one 20–40-year-old men with only a single risk factor meeting a diagnostic criterion, a low level of fasting plasma HDL-cholesterol was found in 27% of them and an elevated level of fasting plasma triglycerides was found in 26%. IFG, found in 6% of these men, was the least prevalent single risk factor meeting a criterion value. Among men older than 40 years meeting a single diagnostic criterion value, HBP, found in 31% of them, was the most prevalent, followed by increased waist circumference, found in 25% of these men, and by elevated fasting plasma triglycerides, found in 23% of them (Table 4).

Increased waist circumference was the most prevalent single risk factor meeting a criterion value in both the 123 younger and the 101 older women, found in 50 and 73%, respectively. We also found that low HDL-cholesterol was the single positive risk factor in 37% of women younger than 40 years with one positive risk factor for the MS.

#### Differences in risk factor prevalence according to sex

Among subjects older than 40 years, men had a significantly higher prevalence of the MS (34%) than women (24%; *p* < 0.05; Table 2). This finding differs from that of Ford et al. (2004) who found a similar prevalence in men and women. However, this sex-associated difference was not found in subjects 20–40 years old, among whom 8% of the men and 5% of the women met criteria for a diagnosis of the MS.

Among women of both age groups, and among 20–40-year-old men, the most commonly encountered diagnostic triad consisted of increased waist circumference, low levels of fasting plasma HDL-cholesterol, and elevated levels of fasting plasma triglycerides (Table 3). This diagnostic triad was found in 76% of the 20–40-year-old women and in 48% of the women older than 40 years who had the MS and was also found in 52% of the men 20–40 years old with the MS. The triads that included increased waist circumference accounted for the diagnosis of the MS in 79% of men older than 40 years.

The prevalence of the 16 possible phenotypes of the MS varied markedly by sex. No single phenotype predominated in the men, whereas nearly one-half of the women had dyslipidemia with increased waist circumference. Among subjects with the MS older than 40 years, women were 3.5 times as likely to have increased waist circumference with dyslipidemia as men.

IFG was the least commonly encountered positive risk factor. Among subjects in the 20–40-year-old age group who met three or more diagnostic criteria for the MS, nine of 33 men and six of 23 women had IFG. Among older subjects who met three or more diagnostic criteria for the MS, 59 of 102 men and 39 of 80 women had IFG. In younger subjects, two of the six possible triads that include IFG, were identified in women, and three in men. The diagnostic triad of IFG, HBP, and low level of fasting plasma HDL-cholesterol was met by none of the men or women in either age groups, and is the only one of the ten possible diagnostic triads not satisfied by any of the 142 subjects who met only three of the five criteria for a diagnosis of the MS. Of the 71 subjects who met four of the five criteria, the cluster of IFG, HBP and low HDL-cholesterol was found in only nine of them.

The distributions of the ten dyads for any two of the five risk factors that met diagnostic criteria for MS differed significantly between men and women (Fig. 2; Table 4). Of the 20–40 year old subjects with two risk factors that met criteria for the MS, 32% of the men but only 17% of the women had elevated levels of fasting plasma triglycerides and low levels of fasting plasma HDL-cholesterol, while 9% of the men, but 64% of the women in this age group, had increased waist circumference and low levels of fasting plasma HDL cholesterol. Among subjects older than 40 years with two positive risk factors, 33% of the men and 31% of the women had increased waist circumference and HBP. IFG was the least likely condition to be encountered in both sexes and in both age groups. Among the 209 subjects in both age groups who met only two diagnostic criteria, 29 of the 111 men and 8 of the 98 women had fasting plasma glucose levels that exceeded the ADA criterion value of 100 mg/dl (Table 4).

Among the subjects with just one risk factor that exceeded a criterion value, there are several notable differences. In 20–40 year old subjects, men were more nearly four times more likely than women to have increased triglycerides (26 vs. 7%) and ten times more likely that women to have HBP (20 vs. 2%). On the other hand women were more than twice as likely as men to have increased waist circumference (50 vs. 21%) and 1.4 times as likely to have low HDL-cholesterol (37 vs. 27%).

In subjects over 40 years with just one positive risk factor, the men were three times more likely than the women to have high fasting plasma triglycerides (23 vs. 8%) or HPB (31 vs. 9%), whereas the women were nearly three times more likely than the men to have increased waist circumference (73 vs. 25%).

## Discussion

Reaven (1988) reported a clustering of dyslipidemia, hypertension, and glucose intolerance which he named syndrome X. The NCEP ATP III expanded the cluster of risk factors for the syndrome by adding waist circumference, a proxy for visceral fat accumulation (Adult Treatment Panel III 2001). The existence of the MS as a true syndrome rather than an association of risk factors that co-vary with obesity has been debated in the literature (Kahn et al. 2005; Cheng and Leiter 2006). In our present analysis of a large quantity of serial metabolic and anthropometric data, subjects without increased waist circumference who met just one or two of the other four criterion values for the MS show the same rate of progression toward a diagnosis of the MS as those subjects with increased waist circumference (Fig. 1). This observation indicates that the risk factors for operate in concert with or without obesity, and implies the existence of a true syndrome that is not simply a collection of covariates driven by obesity.

We found that the prevalence of the MS in both men and women older than 40 years is four times that in 20–40 year-old-subjects. Meigs et al. (2003) also reported substantial age differences in the prevalence of the MS. Among subjects older than 40 years, we found that 24% of the women and 34% of the men met criteria for a diagnosis of the MS. The 34% prevalence that we observed among the men is greater than the 24% reported by Ford et al. (2002). This discrepancy may reflect the difference between men in the FLS who reside predominantly in southwestern Ohio and men in the NHANES US population-based sample studied by Ford et al. (2002).

Our analysis also shows that several of the five risk factors for the MS cluster together as a phenotype more often than others and are the main contributors to the high prevalence of the syndrome. The two most commonly encountered diagnostic triads in the FLS population include increased waist circumference and high fasting plasma triglycerides, coupled with either low fasting plasma HDL-cholesterol or HBP. We found that the frequency distributions of the clusters of risk factors that define the MS depend on both age and sex (Figs. 4 and 5). Older subjects had a greater number of risk factors that exceed criterion values than younger adults among both the men and the women, and older adults were six times more likely than younger adults to meet the diagnostic criterion for HBP and five times more likely to meet the diagnostic criterion for IFG.

Even after we lowered the diagnostic criterion for impaired fasting plasma glucose from 110 mg/dl (NCEP ATP III) to 100 mg/dl (Genuth et al. 2003), this risk factor remained the least commonly met criterion. Only 111 of the 471 men and 58 of the 503 women in our study population had at least one recorded value of fasting plasma glucose >100 mg/dl. Of the six possible diagnostic triads that include IFG, the triad of increased waist circumference, low level of fasting plasma HDL, and IFG applied to only 6 of the 63 women in both age groups combined who met criteria for one of the diagnostic triads. The triad of increased waist circumference, HBP and IFG, and the triad of increased waist circumference, increased level of fasting plasma triglycerides and IFG applied to only four and five, respectively, of the 63 women in both age groups combined who met criteria for one of the diagnostic triads. None of the 63 women met the criteria for the remaining three diagnostic triads that include IFG (Table 3). In contrast to the situation among the women, at least one of the 79 men in both age groups combined met criteria for five of the six diagnostic triads that include IFG.

Ford et al. (2002) also found in the NHANES data set that. IFG was the least commonly met criterion for the MS, especially among women. The FLS data show that 59% of the younger men and 25% of the older men did not meet the diagnostic criteria for any of the five risk factors for the MS. This age dimorphism is also seen in women, among whom 57% of the young women and 27% of the older women did not meet any of the five criteria. It appears that the factors in young women that offer protection against the onset of the MS wane in influence after age 40 years, perhaps reflecting the onset of menopause. In women of both age groups, waist circumference was the risk factor that most frequently exceeded its ATP III criterion value. In sharp contrast, blood pressure was the risk factor that most frequently exceeded its criterion value in older men and, among younger men, the level of fasting plasma HDL-cholesterol was the risk factor that most frequently exceeded its criterion value.

*In view of the age-related and sex-related heterogeneity of the 16 clusters of risk factors that define the MS, it would appear to be clinically important to become aware of the most prevalent clusters of risk factors for the MS in younger and older men and women. We found that the clusters of risk factors that define the MS differ markedly by sex* (Figs. 4 and 5). This phenotypic sexual dimorphism may reflect genetic and/or hormonal influences on adipose tissue, lipid metabolism, glucose metabolism, and blood pressure.

Identification of the onset of the atherogenic profile in men and women according to age is a significant contribution of our analysis of serial data in 974 subjects in the FLS. Identification of the dyad of decreased HDL-cholesterol and increased triglycerides and of the triad that includes diagnostic values for these two risk factors along with HBP in any decade of life, but especially in earlier decades, would appear to be important in regard to predicting the onset of atherosclerotic cardiovascular disease and prescribing preventive regimens.

Family history also affects components of the MS. Arya et al. (2002) reported genetic susceptibility to the MS in Mexican Americans. Data from the Bogalusa Heart Study (BHS) indicate that offspring of parents with coronary artery disease develop excess body fat in childhood and manifest hyperinsulinemia in young adulthood. Other data from the BHS show that clusters of three, four or five risk factors are more likely than would be expected by chance to be inherited by offspring from their parents than one or two risk factors (Berenson and Srinivasan 2001). This covariance of the five risk factors is also salient in our data. Figure 1 shows that within both age groups the values for each of the five risk factors approaches its diagnostic criterion in direct proportion to the number of risk factors meeting diagnostic criteria, from zero to three or more. This observation supports the concept that the MS is a single physiologic entity rather than a collection of independent risk factors.

The 16 different phenotypes of the MS may carry different prognostic implications in regard to the development of type 2 diabetes and cardiovascular disease. For example, phenotypes that include HBP, IFG, and low fasting plasma HDL-cholesterol may have worse prognostic implications than those that include increased waist circumference, elevated fasting plasma triglycerides and low fasting plasma HDL-cholesterol. A priori, it would appear that men and women who met diagnostic criteria for the MS in their third or fourth decade are at greater risk of developing type 2 diabetes or cardiovascular disease later in life than those who failed to meet diagnostic criteria until their fifth or sixth decade.

Our findings will remind clinicians that there are 16 different sets of criteria that trigger the diagnosis of the metabolic syndrome and, therefore, 16 phenotypes are all recognized as the metabolic syndrome. This will be of interest to clinicians who may not have thought about the number of ways five risk factors can be combined into sets of three, four or five and who may not have realized the extent of the clinical variety that exists within the metabolic syndrome.

Since the 16 phenotypes differ markedly in prevalence by age and sex, they may call for different diagnostic and management strategies. Emphasizing age-specific and sex-specific phenotypes should help clinicians identify subjects at highest risk for certain risk factors according to age and sex. We found that the prevalence of the MS was four times greater in the older than in the younger age group. Men and women older than 40 years with the MS also had a significantly greater number of risk factors that exceed diagnostic criterion values than men and women with the MS who are 20–40 years old.

Our analyses are, based on long-term serial observations and should improve the clinical management of obese patients who may or may not also have dyslipidemia, or who may or may not have HBP, and who may or may not have IFG. Our observations should also stimulate clinical investigators and epidemiologists to ascertain what factors determine the striking sex- and age-specificity of the MS phenotypes. Another observation of our study is that the risk factors for the metabolic syndrome cluster together in the absence of increased waist circumference as well as in its presence, indicating that obesity is not the only driver of the metabolic syndrome. Results of our study will provide a basis for therapeutic and preventive strategies to interrupt the pathophysiologic pathway leading to the metabolic syndrome in subjects who advance from meeting none of the criteria for the metabolic syndrome to meeting one or more criteria. In this regard, a sentinel finding of our study is that when one of the five risk factors meets its criterion value, the other four risk factors move in concert closer toward their own criterion values. This phenomenon is also observed when increased waist circumference, a proxy for visceral obesity, is removed from the set of five risk factors. These findings will be of interest to those who follow the literature on the metabolic syndrome because the findings imply that the metabolic syndrome is a true syndromic entity, rather than groups of risk factors that co-vary with obesity.

Our study provides clinicians and investigators with intriguing observations that will sharpen their clinical acumen and should stimulate further research. For example, not one of the 238 subjects with the metabolic syndrome exhibited the diagnostic triad of hypertension, impaired fasting plasma glucose, and low HDL-cholesterol. Therefore, clinicians need not spend time hunting for that particular elusive set of positive risk factors. However, investigators should be keen to discover why this singular set of risk factors is so rare.

At the other extreme of the spectrum of prevalence, 54 of the 238 subjects diagnosed with the metabolic syndrome fit the phenotype of increased waist circumference, low HDL-cholesterol, and hypertriglyceridemia. This prevalent phenotype should spring to mind when one risk factor of the triad is found to be positive. Clinical investigators should also be interested in finding out why this particular set of three risk factors appears so frequently, especially in the younger group of subjects, in whom this diagnostic triad was encountered twice as often as all of the other diagnostic triads combined.

Since the phenotypes of the MS will differ among subjects who meet one of the 16 sets of three, four or five diagnostic criteria, clinicians may need to tailor their preventive and therapeutic strategies to individual subjects who may have phenotypes that carry different clinical implications. Our observation showing that the mean values for each of the five risk factors get progressively worse as the number of risk factors meeting diagnostic criteria increases implies that subjects should be screened routinely to identify the appearance of a single positive risk factor in order to apply interventions to prevent progression to one of the phenotypes of the metabolic syndrome.

Our observations underscore the diversity of the 16 combinations of risk factors that prompt a diagnosis of the metabolic syndrome and highlight the complexity of the interactions between obesity, insulin resistance, dyslipidemia, and hypertension. These observations should stimulate investigators to apply hierarchical modeling and systems analysis to elucidate the clinical implications and long-term outcomes of the metabolic syndrome’s 16 phenotypes.
